# New Definition of Light Chain Monoclonal Gammopathy of Undetermined Significance

**DOI:** 10.1001/jamaoncol.2025.1285

**Published:** 2025-05-29

**Authors:** Thorir Einarsson Long, Saemundur Rognvaldsson, Sigrun Thorsteinsdottir, Ingigerdur Solveig Sverrisdottir, Elias Eythorsson, Jon Thorir Oskarsson, Olafur Skuli Indridason, Runolfur Palsson, Thor Aspelund, Brynjar Vidarsson, Pall Torfi Onundarson, Bjarni Agnar Agnarsson, Margret Sigurdardottir, Ingunn Thorsteinsdottir, Isleifur Olafsson, Asdis Rosa Thordardottir, Asbjorn Jonsson, Gauti Gislason, Andri Olafsson, Malin Hultcrantz, Brian G. M. Durie, Stephen Harding, Thorvardur Jon Love, Ola Landgren, Sigurdur Yngvi Kristinsson

**Affiliations:** 1 University of Iceland, Reykjavik, Iceland; 2 Skane University Hospital, Lund, Sweden; 3 Lund University, Lund, Sweden; 4 Landspitali University Hospital, Reykjavik, Iceland; 5 Rigshospitalet, Copenhagen, Denmark; 6 Sahlgrenska University Hospital, Gothenburg, Sweden; 7 Akureyri Hospital, Akureyri, Iceland; 8 Memorial Sloan Kettering Cancer Center, New York, New York; 9 Cedars-Sinai Outpatient Cancer Center, Los Angeles, California; 10 Binding Site Group Ltd, Birmingham, United Kingdom; 11 Division of Myeloma, Department of Medicine, University of Miami, Sylvester Comprehensive Cancer Center, Miami, Florida

## Abstract

**Importance:**

Recent studies suggest standard reference intervals for serum free light chains (FLC) are inaccurate and that this problem can only be partially remedied by using separate reference intervals for individuals with impaired kidney function. This decreases the utility of FLC testing in the clinical evaluation and follow-up of plasma cell disorders, particularly affecting the diagnosis of light chain (LC) monoclonal gammopathy of undetermined significance (MGUS).

**Objective:**

To evaluate the distribution of serum FLC and FLC ratios in individuals with preserved kidney function and to propose revised reference intervals and a new definition of LC-MGUS.

**Design, Setting, and Participants:**

The Iceland Screens, Treats or Prevents Multiple Myeloma (iStopMM) study is a nationwide prospective study of 75 422 participants (more than 50% of the Icelandic population) 40 years and older who were screened for MGUS. Data were collected from September 2016 to May 2023, and data were analyzed from June 2023 to May 2024.

**Exposure:**

Samples were analyzed by serum protein electrophoresis and immunofixation electrophoresis and FLC assay. Participants were actively followed up for progression.

**Main Outcomes and Measures:**

The rate of abnormal FLC results using standard reference intervals was assessed, and revised age-stratified 99% reference intervals were calculated using nonparametric regression. The prevalence of LC-MGUS based on standard and revised reference intervals was evaluated along with progression to lymphoproliferative disorders.

**Results:**

In total, 41 882 participants met inclusion criteria; a total of 23 786 (56.8%) were female, and the median (IQR) age was 60 (52-68) years. Using standard FLC reference intervals, 7316 κ FLC (17.5%), 1668 λ FLC (4.0%), and 1543 FLC ratios (3.7%) were abnormal. Revised reference intervals were calculated for those younger than 70 years (κ FLC, 6.3-39.0 mg/L; λ FLC, 5.9-36.7 mg/L; FLC ratio, 0.44-2.16) and 70 years or older (κ FLC, 7.0-55.8 mg/L; λ FLC, 6.4-48.0 mg/L; FLC ratio, 0.46-2.59). The prevalence of LC-MGUS was 1.54% (95% CI, 1.46-1.63) using standard intervals and 0.27% (95% CI, 0.23-0.30) using the revised intervals, yielding a decrease of 82%. None of the 1006 persons meeting LC-MGUS criteria based on standard intervals but not based on revised intervals progressed to a lymphoproliferative disorder during a median (range) follow-up of 4.6 (2.5-6.7) years.

**Conclusions and Relevance:**

In this study, a new definition of LC-MGUS based on revised, more accurate FLC reference intervals decreased the false-positive rate of FLC testing by 82%.

## Key Points

**Question:**

Can the definition of light chain (LC) monoclonal gammopathy of undetermined significance (MGUS) be improved?

**Findings:**

In this cohort study of 75 422 individuals, standard reference intervals led to overdiagnosis of monoclonal gammopathies. Revised age-stratified reference intervals for FLC were calculated and a new definition of LC-MGUS proposed; the new definition decreased the prevalence of LC-MGUS by 82%.

**Meaning:**

A new definition of LC-MGUS can substantially decrease false-positive diagnosis and optimize care.

## Teaser

This cohort study evaluates the distribution of serum free light chains and free light chain ratios in individuals with preserved kidney function and proposes revised reference intervals and a new definition of light chain monoclonal gammopathy of undetermined significance.

## Introduction

Monoclonal gammopathy of undetermined significance (MGUS), the precursor of multiple myeloma and related lymphoproliferative disorders,^1,2^ affects 4.2% of the general population older than 50 years.^3,4^ The disorder is characterized by the presence of monoclonal immunoglobulins on serum protein electrophoresis (SPEP) or immunofixation electrophoresis (IFE).^5^ Light chain (LC) MGUS is defined as an abnormal free LC (FLC) ratio caused by elevation of the involved FLC.^4^ The diagnosis of MGUS necessitates further investigations and lifetime follow-up for progression.^5^ Consequently, the accuracy of FLC reference intervals and diagnostic criteria for LC-MGUS is of major importance.

Serum κ and λ FLC and the κ to λ FLC ratio are important for the diagnosis and management of virtually all plasma cell disorders.^6^ Existing reference intervals were derived from a relatively small cohort (n = 282) of blood donors.^7^ The reference intervals were determined as central 95% for absolute κ FLC and λ FLC, while 100% (FLC ratio of 0.26 to 1.65) was suggested as a diagnostic interval for the FLC ratio. Recent evidence suggests that existing reference intervals may not accurately represent the distribution of FLC in the general population.^8,9,10,11,12^ This discrepancy can partly be attributed to the influence of reduced kidney function on FLC levels. Our group has recently redefined the reference intervals for FLC in individuals with reduced kidney function, defined as an estimated glomerular filtration rate (eGFR) less than 60 mL/min/1.73 m^2^.^13^ However, emerging evidence suggests that standard reference intervals for FLC are inaccurate even among individuals with preserved kidney function (eGFR of 60 mL/min/1.73 m^2^ or greater).^8,10,11,14^ This could lead to overdiagnosis, unnecessary follow-up, and a distorted evaluation of treatment responses.

To evaluate this, we assessed the distribution of serum κ FLC, λ FLC, and the FLC ratio in the Iceland Screens, Treats or Prevents Multiple Myeloma (iStopMM) study cohort, a population-based prospective cohort of 75 422 persons screened with SPEP, IFE, and FLC assays. Our aim was to describe the rate of abnormal findings using the standard FLC reference intervals in individuals with preserved kidney function and to define revised reference intervals if the data show that the standard definition leads to excessive number of individuals being classified as having abnormal finding, yielding a new definition of LC-MGUS.

## Methods

The iStopMM study (NCT03327597) is a nationwide, prospective study of MGUS inviting all registered residents of Iceland born in 1975 or earlier who were alive on September 9, 2016, to participate. Approvals from the Icelandic National Bioethics Committee and the Data Protection Authority in Iceland were obtained. All participants provided written informed consent. This study followed the Strengthening the Reporting of Observational Studies in Epidemiology (STROBE) reporting guideline. The study design has been described in detail elsewhere.^15^

### Data Collection

Study participants were screened with SPEP, IFE, and serum FLC testing, and those with MGUS were randomized into a clinical trial of work-up and follow-up (eMethods in Supplement 1). Serum creatinine measurements were retrieved from a central laboratory database, and the eGFR calculated using the Chronic Kidney Disease Epidemiology Collaboration equation.^16^ Participants’ previous diagnosis codes were acquired from the Icelandic Directorate of Health. The reliability of disease diagnoses in the register has been validated.^17^

### Definitions

LC-MGUS was defined as an FLC ratio outside the reference interval with an increase in the involved κ or λ FLC, without evidence of M protein on SPEP or IFE and in absence of myeloma defining events.^4,5^ MGUS was defined as M protein detected on SPEP or IFE. The involved FLC was the one with the higher absolute serum concentration. The standard FLC reference intervals (FLC ratio of 0.26-1.65; κ FLC range, 3.3 to 19.4 mg/L; and λ FLC range, 5.7 to 26.3 mg/L) were used in persons with eGFR of 60 mL/min/1.73 m^2^ or greater and the previously published iStopMM kidney reference intervals for persons with eGFR less than 60 mL/min/1.73 m^2^.^7,13^ Preserved kidney function was defined as an eGFR of 60 mL/min/1.73 m^2^ or greater.

### Exclusion Criteria

Individuals with known lymphoproliferative disorders at screening and those with MGUS or an involved/uninvolved FLC ratio of 100 or greater were excluded.^5^ Participants with eGFR less than 60 mL/min/1.73 m^2^ or without available serum creatinine measurement within 1 year before or after screening were also excluded.

### Statistical Analysis

Reference intervals were defined according to standard guidelines from the International Federation of Clinical Chemistry.^18^ The 0.5, 2.5, 97.5, and 99.5 percentiles of κ FLC, λ FLC, and the FLC ratio distributions were assessed in general and for subgroups based on age, sex, and eGFR. Direct visualization and the Anderson-Darling test were used to assess normality of data. Reference intervals were determined using nonparametric regression and bootstrapping applied to calculate the 95% CIs. The Horn algorithm was used to assess outliers, but none were removed from the analysis (eMethods in Supplement 1). Partitioning was determined based on the proportion of subgroups (age, sex, and eGFR) outside the whole-group reference interval. If the analysis resulted in a subgroup placed outside the central 99% reference interval with a rate of abnormal findings that exceeded 0.8% or was below 0.2%, the reference interval was partitioned.^19^ Central 95% and 99% reference intervals were calculated for serum κ and λ FLC and the FLC ratio for the entire cohort, as well as for subgroups based on age, sex, and eGFR. First, a single central 99% reference interval was constructed for κ and λ FLC and the FLC ratio for all participants.^13^ A significant proportion of the age group 70 years and older was observed to fall outside the common whole-group reference intervals (eTable 1 in Supplement 1). As values were highly dependent on age, the reference intervals were partitioned by age younger than 70 years and 70 years and older. After partitioning the reference intervals, the proportion of the age, sex, and eGFR subgroups with values outside the reference intervals was deemed appropriate (eTable 2 in Supplement 1).

The prevalence of LC-MGUS was estimated for the total screened iStopMM cohort (N = 75 422), using both the standard reference intervals and the revised reference intervals. For those with impaired kidney function, revised reference intervals previously published by our group were used.^13^ For those with preserved kidney function, the revised reference intervals described in this article were used. When eGFR within 1 year of screening was not available for the prevalence calculation, the eGFR value closest to screening (n = 16 654) was used when available and missing eGFR values (n = 6248) were accounted for using predictive mean matching multiple imputation. The age-specific and sex-specific prevalence was determined and visualized using a fitted binomial function adjusted for age and sex and their interaction (eMethods in Supplement 1). The standard error of the fitted model was used to compute the 95% CIs for the prevalence.

The association between serum κ FLC, λ FLC, and FLC ratio with age and eGFR was assessed graphically by plotting 4-knot restricted cubic splines. Correlation was assessed using the Spearman correlation coefficient. A Student *t* test was used to calculate a 2-tailed *P* value for the correlation, where a value less than .05 was considered statistically significant. Data were collected from September 2016 to May 2023, and data were analyzed from June 2023 to May 2024. All statistical analysis and data visualization were performed in R version 4.2.0 (The R Foundation).^20,21,22,23,24,25,26,27,28,29^

## Results

Of the 148 704 persons invited to participate, 80 758 (54.3%) provided written informed consent and 75 422 (93%) of those consented were screened with SPEP and FLC measurement. After excluding individuals with positive IFE findings (n = 3614), eGFR less than 60 mL/min/1.73 m^2^ (n = 6568), no available serum creatinine measurement within 1 year of screening (n = 22 902), or a known lymphoproliferative disorder (n = 456) (eTable 4 in Supplement 1), 41 882 participants were eligible for the analysis; a total of 23 786 (56.8%) were female, and the median (IQR) age was 60 (52-68) years. The median (IQR) eGFR was 84 (74-94) mL/min/1.73 m^2^. The median (IQR) serum κ FLC was 14.3 (11.6-17.8) mg/L; serum λ FLC, 14.2 (11.6-17.5) mg/L; and FLC ratio, 1.02 (0.85-1.21) (Table 1).

**Table 1.** Characteristics of the Study Cohort and Median Levels of Serum κ and λ Free Light Chain (FLC) and the FLC Ratio, Categorized According to Sex and Levels of Estimated Glomerular Filtration Rate (eGFR)

Characteristic	No. (%)		
	Overall	Age <70 y	Age ≥70 y
Total, No.	41 882	34 195	7687
Age, median (IQR), y	60 (52-68)	57 (51-63)	75 (73-79)
Sex			
Female	23 786 (56.8)	19 814 (57.9)	3972 (51.7)
Male	18 096 (43.2)	14 381 (42.1)	3715 (48.3)
Race and ethnicity, No./total No. (%)^a^			
Asian	4/2303 (0.2)	4/1209 (0.3)	0/1094
Black	2/2303 (<0.1)	1/1209 (0.1)	1/1094 (<0.1)
Hispanic	1/2303 (<0.1)	1/1209 (0.1)	0/1094
White	2296/2303 (99.7)	1203/1209 (99.5)	1093/1094 (99.9)
eGFR, median (IQR), mL/min/1.73 m^2^	84 (74-94)	87 (76-96)	76 (68-84)
κ FLC, median (IQR), mg/L	14.3 (11.6-17.8)	13.8 (11.3-17.0)	16.6 (13.3-21.0)
Sex, median (IQR)			
Male	14.8 (12.0-18.4)	14.1 (11.6-17.4)	17.4 (14.0-22.0)
Female	13.9 (11.3-17.2)	13.6 (11.1-16.7)	15.7 (12.6-19.7)
eGFR, median (IQR), mL/min/1.73 m^2^			
90-120	13.2 (10.8-16.2)	13.2 (10.8-16.1)	14.1 (11.7-18.3)
60-89	14.9 (12.1-18.5)	14.3 (11.7-17.6)	16.6 (13.4-21.0)
λ FLC, median (IQR), mg/L	14.2 (11.6-17.5)	14.0 (11.4-17.1)	15.5 (12.5-19.2)
Sex, median (IQR)			
Male	14.3 (11.7-17.6)	13.9 (11.4-17.0)	15.8 (12.9-19.8)
Female	14.1 (11.5-17.3)	14.0 (11.4-17.1)	14.9 (12.0-18.6)
eGFR, median (IQR), mL/min/1.73 m^2^			
90-120	13.7 (11.2-16.8)	13.7 (11.2-16.7)	14.6 (11.8-18.1)
60-89	14.5 (11.8-17.8)	14.1 (11.6-17.2)	15.4 (12.5-19.2)
FLC ratio, median (IQR)	1.02 (0.85-1.21)	1.00 (0.84-1.19)	1.09 (0.90-1.30)
Sex, median (IQR)			
Male	1.04 (0.88-1.24)	1.03 (0.86-1.22)	1.11 (0.92-1.32)
Female	1.00 (0.83-1.19)	0.98 (0.82-1.17)	1.06 (0.88-1.27)
eGFR, median (IQR), mL/min/1.73 m^2^			
90-120	0.97 (0.81-1.16)	0.97 (0.81-1.16)	1.00 (0.81-1.22)
60-89	1.04 (0.87-1.24)	1.02 (0.86-1.22)	1.09 (0.91-1.30)
Comorbidities			
Hypertension	15 387 (36.7)	10 931 (32.0)	4456 (58.0)
Diabetes	3108 (7.4)	2263 (6.6)	845 (11.0)
Ischemic heart disease	4355 (10.4)	2506 (7.3)	1849 (24.1)
Heart failure	1073 (2.6)	501 (1.5)	572 (7.4)
Cardiac arrythmia	4406 (10.5)	2722 (8.0)	1684 (21.9)
Peripheral artery disease	1120 (2.7)	676 (2.0)	444 (5.8)
Obesity	4528 (10.8)	3930 (11.5)	598 (7.8)
Endocrine disorder (nondiabetic)	5696 (13.6)	4492 (13.1)	1204 (15.7)
Chronic lung disease	12 794 (30.5)	9926 (29.0)	2868 (37.3)
Liver disease	902 (2.2)	717 (2.1)	185 (2.4)
Malignancy	4282 (10.2)	2765 (8.1)	1517 (19.7)
Neurological disease	1768 (4.2)	1322 (3.9)	446 (5.8)

^a^ Information on self-reported race and ethnicity only available for persons with monoclonal gammopathy of undetermined significance randomized to study arms 2 and 3, which were excluded from the determination of FLC reference intervals part of the study.

The serum FLC increased significantly with age, with a positive correlation between age and serum κ FLC (ρ = 0.27; *P* < .001) and λ FLC (ρ = 0.14; *P* < .001). A negative correlation was observed between eGFR and κ FLC (ρ = −0.24; *P* < .001) and λ FLC (ρ = −0.12; *P* < .001). The association between κ FLC, λ FLC, and the FLC ratio and age and eGFR is demonstrated in the eFigure in Supplement 1.

### Performance of Standard Reference Intervals

**Figure 1.**
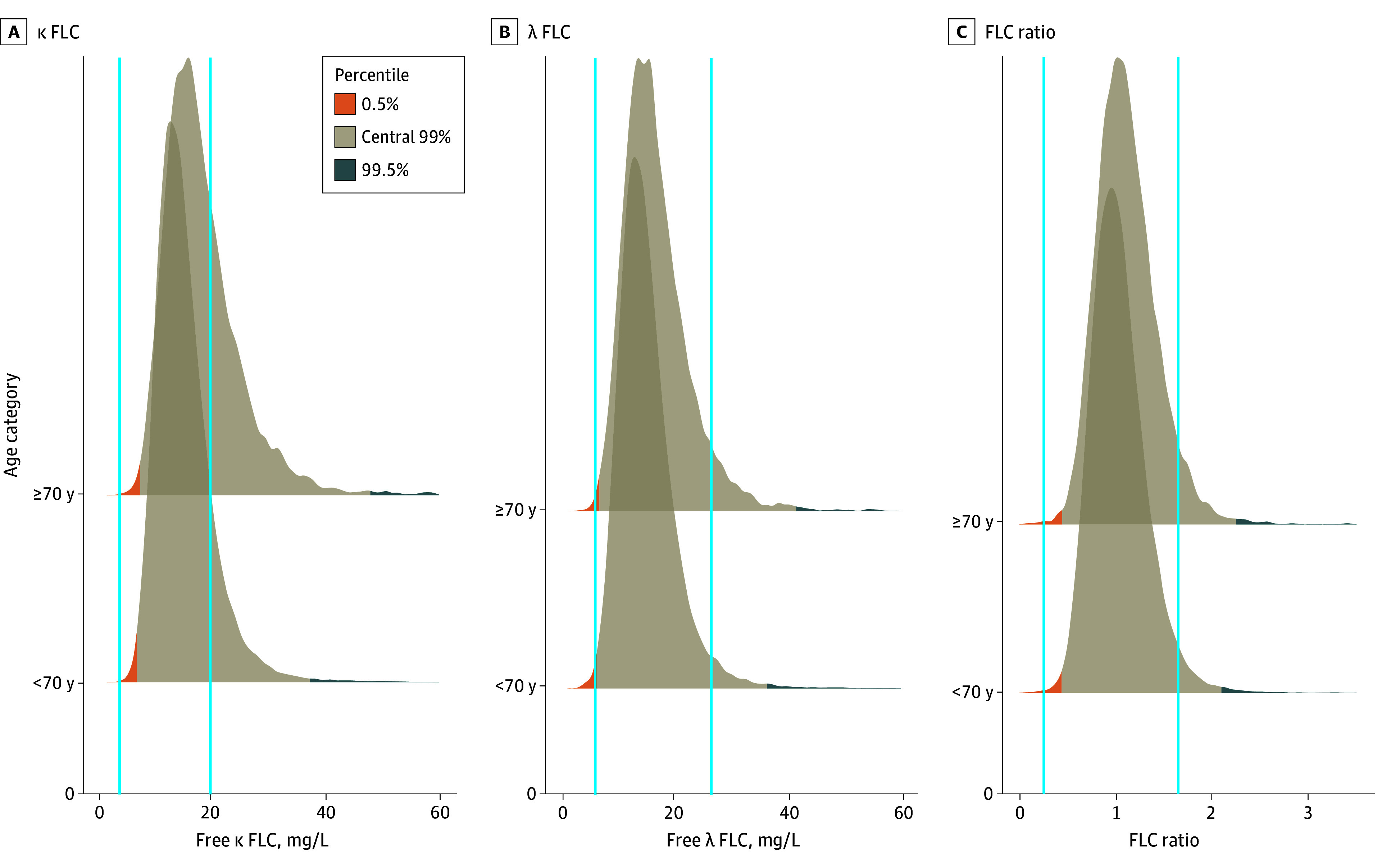
Distribution of κ and λ Free Light Chains (FLC) and FLC Ratios in Individuals With Estimated Glomerular Filtration Rate of 60 mL/min/1.73 m^2^ or Greater Compared With Standard Reference Intervals by Age Categories A, Serum κ FLC (vertical blue lines, 3.3-19.4 mg/L). B, Serum λ FLC (vertical blue lines, 5.7-26.3 mg/L). C, FLC ratio (vertical blue lines, 0.26-1.65). κ and λ FLC are truncated at 60 mg/L and the FLC ratio at 3.5 in the figure panels for better visualization.

Standard reference intervals yielded a high rate of aberrant results in the cohort (Figure 1), with 17.5% of κ FLC, 4.0% of λ FLC, and 3.7% of the FLC ratios classified as abnormal. The distribution of abnormal results was predominantly above the upper limit of normal for all 3 parameters, with 17.5% (n = 7313) having high κ FLC and 0.01% (n = 3) low κ FLC, 3.7% (n = 1525) having high λ FLC and 0.3% (n = 143) low λ FLC , and 3.6% (n = 1505) having a high FLC ratio and 0.1% (n = 38) a low ratio. The crude rate of LC-MGUS using the standard reference intervals in this subpopulation with preserved kidney function was 2.0% (n = 850), of whom 813 had κ LC-MGUS and 37 had λ LC-MGUS. Combining the standard reference intervals for κ FLC, λ FLC, and FLC ratio in those with eGFR of 60 mL/min/1.73 m^2^ or greater with the iStopMM kidney reference intervals for individuals with eGFR less than 60 mL/min/1.73 m^2^, the whole-group prevalence of LC-MGUS was 1.54% (95% CI, 1.46-1.63). The prevalence of κ LC-MGUS was 1.47% (95% CI, 1.39-1.55), and the prevalence of λ LC-MGUS was 0.07% (95% CI, 0.05-0.09).

### Revised Reference Intervals and New Definition of LC-MGUS

**Figure 2.**
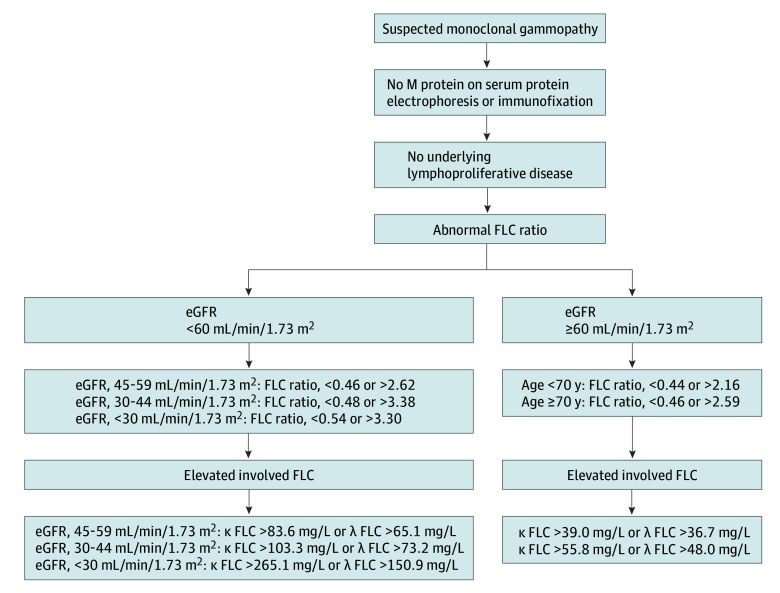
New Definition of Light Chain Monoclonal Gammopathy of Undetermined Significance Reference intervals for estimated glomerular filtration rate (eGFR) less than 60 mL/min/1.73 m^2^ have been previously published.^13^ Involved free light chain (FLC) defined as high λ FLC with abnormally low FLC ratio and high κ FLC with elevated FLC ratio.

**Figure 3.**
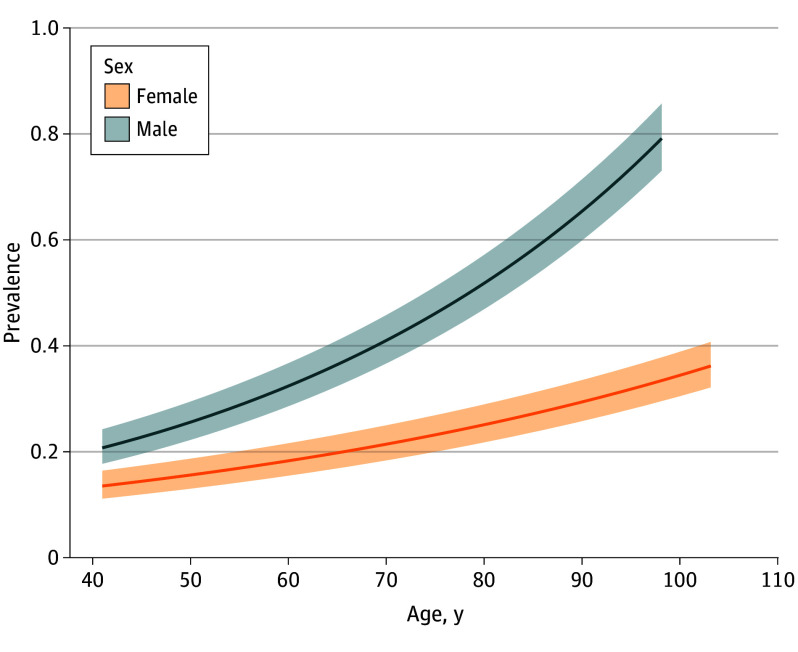
Prevalence of Light Chain Monoclonal Gammopathy of Undetermined Significance Using the New Definition by Age and Sex The shaded area indicates 95% CIs.

The revised central 99% reference intervals for κ FLC, λ FLC, and FLC ratio are shown in Table 2. Revised central 95% reference intervals for both κ and λ FLC and the FLC ratio were also determined and are shown in eTable 3 in Supplement 1. Based on our findings, a new definition of LC-MGUS is introduced in Figure 2. Using this new definition for the whole iStopMM cohort, including persons with eGFR less than 60 mL/min/1.73 m^2^ (n = 8637) and eGFR of 60 mL/min/1.73 m^2^ or greater (n = 66 785), the overall prevalence of LC-MGUS was 0.27% (95% CI, 0.23-0.30). The prevalence of κ LC-MGUS was 0.14% (95% CI, 0.12-0.17), and the prevalence of λ LC-MGUS was 0.12% (95% CI, 0.10-0.15). Among persons 40 years and older, the prevalence of LC-MGUS was 0.36% (95% CI, 0.32-0.40) for male individuals and 0.19% (95% CI, 0.16-0.22) for female individuals. The prevalence of LC-MGUS increased with age and was 0.18% (95% CI, 0.15-0.21) in individuals aged 40 to 49 years and 0.41% (95% CI, 0.37-0.46) in persons 80 years and older (Figure 3). The relative decrease in the prevalence of LC-MGUS using the new definition compared with the combination of the standard and revised kidney criteria was 82%, with a 95% relative decrease in κ LC-MGUS and a 71% relative increase in λ LC-MGUS.

**Table 2.** Revised Reference Intervals for Serum κ Free Light Chain (FLC), λ FLC, and FLC Ratio in Individuals With Preserved Kidney Function Stratified by Age^a^^,^^b^

Age group	Total, No.	FLC (95% CI), mg/L	
		0.5th Percentile	99.5th Percentile
**κ FLC**			
<70 y	33 181	6.3 (6.2-6.4)	39.0 (37.2-40.4)
≥70 y	8701	7.0 (6.7-7.2)	55.8 (52.4-61.0)
**λ FLC**			
<70 y	33 181	5.9 (5.7-6.0)	36.7 (35.8-37.6)
≥70 y	8701	6.4 (6.3-6.6)	48.0 (42.6-53.5)
**FLC ratio**			
<70 y	33 181	0.44 (0.43-0.45)	2.16 (2.09-2.20)
≥70 y	8701	0.46 (0.42-0.49)	2.59 (1.82-2.82)

^a^ Preserved kidney function defined as an estimated glomerular filtration rate of 60 mL/min/1.73 m^2^ or greater.

^b^ Determined as central 99% reference intervals. Standard reference intervals: κ FLC, 3.3-19.4 mg/L; λ FLC, 5.7-26.3 mg/L; and FLC ratio, 0.26-1.65.

### Follow-Up

Of the participants diagnosed with LC-MGUS based on standard reference intervals who did not meet the diagnostic criteria using our revised reference intervals (n = 1006), none had progressed to a lymphoproliferative disorder after 4648 patient-years of follow-up, with a median (range) follow-up time of 4.6 (2.5-6.7) years. Bone marrow biopsy findings were available for 22 persons, with none revealing more than 10% plasma cells as an indication of a more advanced disease than MGUS.

## Discussion

Based on prospective screening of more than 75 000 individuals, we propose a revision of the reference intervals for serum κ FLC, λ FLC and FLC ratio, stratified by age, along with a new definition of LC-MGUS to significantly improve the diagnostic accuracy of this disorder. In the iStopMM cohort, applying our new definition decreased the prevalence of LC-MGUS by 82% in individuals 40 years and older, without any of the previously misdiagnosed cases progressing to a lymphoproliferative disorder during a follow-up period of 4648 patient-years.

In our large prospective screened cohort, we found that the standard reference intervals for κ FLC, λ FLC, and the FLC ratio among individuals with preserved kidney function resulted in marked overdiagnosis of κ LC and underdiagnosis of λ LC monoclonal gammopathies. The revised intervals could facilitate future risk stratification efforts and focus the treatment response criteria on individuals with more clinically relevant disease. In turn, the new definition of LC-MGUS may alleviate the burden of anxiety and unnecessary testing in individuals previously receiving what we can now recognize as false-positive diagnoses.^30,31^ These improvements could significantly decrease unnecessary costs from extensive work-ups and lifelong monitoring, directing limited health care resources to relevant cases.

Given the pivotal role of serum FLC measurement in detection, risk stratification, and monitoring of lymphoproliferative disorders, ensuring the accuracy of their reference intervals is crucial. Our newly proposed reference intervals for κ and λ FLC differ significantly from the standard intervals.^7^ In our cohort, we observed markedly higher levels of κ FLC, λ FLC, and FLC ratio compared with the values reported in the original study. This finding is in line with several recent smaller studies that have consistently demonstrated a high rate of false-positive results in individuals with slightly abnormal κ FLC levels.^8,9,10,12^ These results, together with our current findings, emphasize the imprecision of the standard reference intervals and the need for revised reference intervals. Discrepancies between our and the original reference intervals likely stem from a combination of different baseline populations studied and a change in κ levels of the Freelite assay (The Binding Site) over time.^9,12^ The exact cause of this upward drift in κ levels remains unknown but could possibly be linked to incremental changes in calibration over time. Interestingly, within our revised reference interval, the lower limit of the FLC ratio has significantly increased. This shift cannot be solely attributed to an upward drift in λ FLC levels, as this was minimal compared with the substantial change in κ FLC levels. It appears that the lower limit of the standard reference interval for FLC ratio has hitherto been erroneously low, resulting in false-negative values in cases of λ plasma cell disorders, which is consistent with previous studies.^13,32^ This discrepancy implies that use of the standard reference interval results in overdiagnosis of κ LC disease in persons without a true monoclonal disorder and underdiagnosis of λ LC disease in individuals with an underlying monoclonal disorder.

The prevalence of LC-MGUS was 0.27% (95% CI, 0.23-0.30) in the iStopMM cohort using our updated definition of the disorder, whereas the prevalence was 1.54% (95% CI, 1.46-1.63) using the older definition. Thus, the prevalence derived using the older definition was almost 2-fold higher than the 0.8% rate published by Dispenzieri et al^4^ in 2010, despite the latter study lacking key information on kidney function. A notable difference is the rate of κ to λ LC-MGUS ratio in their study, which was 2.8:1 compared with 22:1 in our study using the same criteria and reference intervals. By contrast, the κ to λ ratio using our new definition of LC-MGUS was 1.2:1. This difference in both prevalence rates and the κ to λ LC-MGUS again highlights the need for updated reference intervals and a new definition of LC-MGUS.

It is noteworthy that after 4648 patient-years of follow-up, no individual had been diagnosed with a lymphoproliferative disorder among those who were considered to have had a false-positive diagnosis of LC-MGUS based on the standard definition. Furthermore, none of the individuals who underwent bone marrow biopsy exhibited abnormal results. This indicates that we are unlikely to miss clinically important lymphoproliferative disorders despite the substantial reduction in overall LC-MGUS cases.

We propose that our new definition of LC-MGUS be implemented in clinical practice guidelines. Furthermore, we propose that our revised reference intervals, stratified by age older than or younger than 70 years in persons with preserved kidney function, be implemented in the clinic for interpretation of FLC values. Possible barriers, such as clinician awareness and delayed guideline updates, need to be addressed through active collaboration and education. Implementation will minimize the rate of false-positive results, thereby avoiding unnecessary testing and diagnostic procedures. In addition, the revised reference intervals will alter and likely optimize both risk stratification models and treatment response criteria. However, it is of utmost importance that the new reference intervals be studied in this setting before being implemented in clinical practice. Regarding frequency of FLC analysis, we recommend adhering to existing clinical guidelines when using the revised reference intervals. However, false-negative results cannot be ruled out particularly in disorders characterized by small pathological clones. Therefore, clinicians should still exercise their clinical judgement, especially in borderline cases when repeated testing in 3 to 6 months may be advisable.

### Strengths and Limitations

A major strength of this study is its extensive high-quality data generated by screening most of the Icelandic population older than 40 years with SPEP, IFE, and FLC. This along with readily available information on kidney function allowed for careful assessment and determination of reference intervals, eliminating the risk of selection bias. Another strength is that all screening measurements were conducted in the same laboratory using consistent methods and lot-to-lot comparisons, thereby minimizing the impact of variability between different assays.

This study has limitations, including the genetic and ethnic homogeneity of the Icelandic study population, which may preclude generalizability of the findings. However, since this definition was initially presented,^33^ it has already been validated in an Israeli cohort,^34^ a Lebanese cohort,^35^ and a younger population of African American and South African individuals.^36^ Furthermore, the serum creatinine measurements were not always carried out at the time of screening, which could theoretically affect our results. However, we believe this effect to be minimal as the study included only participants with preserved kidney function, excluding all serum creatinine measurements obtained more than 1 year from screening. The current study used the Freelite assay for the FLC measurements, and therefore the novel reference intervals should only be used for that assay. Notably, the Freelite assay is the most widely used FLC assay globally, making our results applicable on a large scale.

## Conclusions

In conclusion, using data from, to our knowledge, the largest population-based screening study of monoclonal gammopathies to date, we have demonstrated that standard reference intervals for κ FLC, λ FLC, and the FLC ratio are inaccurate among persons with preserved kidney function, causing high rates of false-positive κ and false-negative λ monoclonal gammopathies. We propose revised reference intervals for κ FLC, λ FLC, and the FLC ratio, stratified for persons younger than 70 years and 70 years and older. Building on our prior findings in individuals with reduced kidney function, we propose a new definition of LC-MGUS. Implementing the new definition decreased the rate of false-positive diagnosis of LC-MGUS by more than 80% in our study cohort. While the consistency of our findings across multiple populations supports their validity,^34,35,36^ further studies in diverse global cohorts, individuals with previous history of lymphoproliferative disorders, as well as their integration into prognostic models and response criteria will be valuable to confirm the broader applicability of these revised reference intervals. These intervals could alleviate unwarranted psychological and financial burdens in the care of individuals with LC gammopathies, improving health care system efficiency and the quality of life for affected individuals.

## Notes

Supplement 1. **eMethods.** Screening for MGUS **eTable 1.** Central 99 Percentiles and 95% CIs for Serum Kappa FLC, Lambda FLC and the FLC Ratio for the Whole Group and Age and Sex Subgroups **eTable 2.** Rate of Abnormal Results in Age, Sex, and eGFR Subgroups When a Revised Reference Interval for Kappa FLC, Lambda FLC, and the FLC Ratio is Used in Persons Below and Above Age 70 Years **eTable 3.** Revised Central 95% Reference Intervals for Serum Kappa FLC, Lambda FLC and FLC Ratio in Individuals With Preserved Kidney Function (eGFR ≥60 mL/min/1.73 m^2^), Stratified by Age **eTable 4.** Characteristics of the Persons Excluded From the Determination of FLC Reference Intervals **eFigure.** Scatterplots Demonstrating the Association of Serum Kappa FLC, Lambda FLC and the FLC Ratio With Age and eGFR

Supplement 2. Data Sharing Statement
