# Supplementary material for: New Definition of Light Chain Monoclonal Gammopathy of Undetermined Significance
Source: JAMA Oncol. 2025 May 29;11(7):753–61. doi: 10.1001/jamaoncol.2025.1285 (PMC12123532; doi:10.1001/jamaoncol.2025.1285)
Supplement: Supplement 1. — eMethods. Screening for MGUS eTable 1. Central 99 Percentiles and 95% CIs for Serum Kappa FLC, Lambda FLC and the FLC Ratio for the Whole Group and Age and Sex Subgroups eTable 2. Rate of Abnormal Results in Age, Sex, and eGFR Subgroups When a Revised Reference Interval for Kappa FLC, Lambda FLC, and the FLC Ratio is Used in Persons Below and Above Age 70 Years eTable 3. Revised Central 95% Reference Intervals for Serum Kappa FLC, Lambda FLC and FLC Ratio in Individuals With Preserved Kidney Function (eGFR ≥60 mL/min/1.73 m2), Stratified by Age eTable 4. Characteristics of the Persons Excluded From the Determination of FLC Reference Intervals eFigure. Scatterplots Demonstrating the Association of Serum Kappa FLC, Lambda FLC and the FLC Ratio With Age and eGFR [file jamaoncol-e251285-s001.pdf]

Supplemental Online Content

Einarsson Long T, Rögnvaldsson S, Thorsteinsdottir S, et al. New definition of light chain monoclonal gammopathy of undetermined significance. *JAMA Oncol*. Published online May 29, 2025. doi:10.1001/jamaoncol.2025.1285

eMethods. Screening for MGUS
eTable 1. Central 99 Percentiles and 95% CIs for Serum Kappa FLC, Lambda FLC and the FLC Ratio for the Whole Group and Age and Sex Subgroups
eTable 2. Rate of Abnormal Results in Age, Sex, and eGFR Subgroups When a Revised Reference Interval for Kappa FLC, Lambda FLC, and the FLC Ratio is Used in Persons Below and Above Age 70 Years
eTable 3. Revised Central 95% Reference Intervals for Serum Kappa FLC, Lambda FLC and FLC Ratio in Individuals With Preserved Kidney Function (eGFR ≥60 mL/min/1.73 m^2), Stratified by Age
eTable 4. Characteristics of the Persons Excluded From the Determination of FLC Reference Intervals
eFigure. Scatterplots Demonstrating the Association of Serum Kappa FLC, Lambda FLC and the FLC Ratio With Age and eGFR

This supplementary material has been provided by the authors to give readers additional information about their work.

© 2025 Einarsson Long T et al. *JAMA Oncology*.

eMethods. Screening for MGUS

Serum samples were collected from study participants and sent to The Binding Site Ltd. in Birmingham, UK for SPEP, IFE, and serum FLC testing. SPEP was performed using capillary zone electrophoresis (CZE; Helena Laboratories, Texas, USA). Quantification of kappa and lambda FLCs was carried out using the FREELITE assay (The Binding Site Ltd.). If the SPEP results indicated monoclonal (M) protein bands or if the FLC values deviated from standard reference intervals,[7] IFE was performed (Helena Laboratories, Texas, USA). Persons who had an abnormal screening test entered a randomized clinical trial with three study arms. Individuals in arm 1 were unaware of their results and continued regular care in the Icelandic health service. Individuals in arms 2 and 3 were invited to a clinical study center for further evaluation and follow-up, including bone marrow sampling from all except those with low-risk MGUS in arm 2 and from all participants in arm 3. Participants in arm 2, excluding low risk of MGUS, and all participants in arm 3 underwent annual follow-up regarding progression to a lymphoproliferative disorder. Individuals with low-risk MGUS in arm 2 were followed every 2-3 years. Detailed information on the analytical methods and follow-up has been published previously.[15]

The determination of the reference intervals for kappa free light chain (FLC), lambda FLC and the FLC ratio was performed using the RefLimit function of the referenceIntervals package in R. The exact command parameters used were the following: Function: RefLimit, value/arguments: out.method="horn“, out.rm=F, RI="n“, CI="boot“, refConf=0.99 ("0.95“ when central 95% reference intervals were determined), limitConf=0.95, bootStat="basic“.

© 2025 Einarsson Long T et al. *JAMA Oncology*.

Missing estimated glomerular filtration rate (eGFR) values were addressed using predictive mean matching (PMM) via the aregImpute function from the *Hmisc* package in R. The imputation model incorporated key covariates including age, sex, hypertension, ischemic heart disease, heart failure, and diabetes to predict missing eGFR values. A total of 20 nearest observed values (nk=20) were used. The distribution of the imputed values were comparable to the observed values. A binomial logistic regression model was fitted using the glm function with a binomial family to examine the relationship between LC-MGUS and predictors, including age and sex as well as interaction between age and sex. This model estimates the log odds of the outcome as a linear function of the predictors and their interactions, providing insights into how these variables influence the probability of the outcome. Following model fitting, predicted probabilities were computed for each observation. To assess the precision of these predicted probabilities, Wilson score intervals were calculated using the *binom* package.

© 2025 Einarsson Long T et al. *JAMA Oncology*.

eTable 1. Central 99 Percentiles and 95% CIs for Serum Kappa FLC, Lambda FLC and the FLC Ratio for the Whole Group and Age and Sex Subgroups

The rate of abnormal results in age, sex, and eGFR subgroups based on a single unified whole-group reference is shown.

Kappa FLC,mg/L							
Group	N	0.5th percentile		99.5th percentile		% below	% above
		Kappa, mg/L	95% CI	Kappa, mg/L	95% CI		
All participants	41,882	**6.4**	6.3 – 6.5	**43.5**	41.8 – 45.3		
Females	23,786	6.2	6.1 – 6.4	38.9	37.0 – 40.5	0.6%	0.3%
Males	18,096	6.6	6.5 – 6.8	48.3	44.6 – 50.9	0.4%	0.7%
Age, years							
40 – 49	7,389	6.0	5.7 – 6.2	34.8	31.2 – 37.1	0.8%	0.2%
50 – 59	12,788	6.2	6.0 – 6.4	36.7	32.9 – 38.9	0.6%	0.3%
60 – 69	13,004	6.6	6.4 – 6.8	42.4	39.6 – 44.9	0.4%	0.4%
70 – 79	6,807	6.9	6.6 – 7.1	52.6	47.0 – 57.9	0.2%	0.8%
≥80	1,894	7.2	6.6 – 7.7	61.6	3.3 – 72.4	0.2%	2.0%
eGFR, mL/min/1.73 m^2							
60-89	27,420	6.8	6.6 – 6.9	46.5	44.4 – 49.2	0.3%	0.6%
≥90	14,462	5.9	5.7 – 6.1	36.5	32.7 – 38.0	0.9%	0.3%
Lambda FLC, mg/L							
Group	N	0.5th percentile		99.5th percentile		% below	% above
		Lambda, mg/L	95% CI	Lambda, mg/L	95% CI		
All participants	41,882	**6.0**	5.9 – 6.1	**39.3**	38.2 – 40.6		
Females	23,786	6.1	5.9 – 6.2	37.0	35.4 – 38.0	0.5%	0.4%
Males	18,096	6.0	5.7 – 6.1	42.6	38.5 – 44.7	0.5%	0.6%
Age, years							
40 – 49	7,389	5.8	5.4 – 6.0	35.6	32.0 – 38.3	0.6%	0.4%
50 – 59	12,788	5.8	5.6 – 6.0	36.2	34.8 – 38.3	0.6%	0.3%
60 – 69	13,004	6.1	5.9 – 6.3	37.9	35.7 – 39.2	0.4%	0.4%
70 – 79	6,807	6.4	6.3 – 6.6	48.0	42.3 – 53.5	0.2%	0.9%
≥80	1,894	6.3	5.6 – 7.2	49.5	44.1 – 58.3	0.4%	1.2%
eGFR, mL/min/1.73 m^2							
60 – 89	27,420	6.2	6.1 – 6.3	39.9	38.1 – 41.4	0.4%	0.5%
≥90	14,462	5.7	5.5 – 6.1	37.6	35.4 – 39.1	0.7%	0.4%
FLC ratio							
Group	N	0.5th percentile		99.5th percentile		% below	% above
		FLC ratio	95% CI	FLC ratio	95% CI		
All participants	41,882	**0.44**	0.43 – 0.45	**2.25**	2.19 – 2.31		
Females	23,786	0.44	0.43 – 0.45	2.15	2.06 – 2.21	0.5%	0.4%
Males	18,096	0.45	0.41 – 0.47	2.36	2.17 – 2.44	0.5%	0.6%

© 2025 Einarsson Long T et al. *JAMA Oncology*.

Age, years							
40 – 49	7,389	0.44	0.40 – 0.45	2.04	1.95 – 2.15	0.5%	0.3%
50 – 59	12,788	0.44	0.41 – 0.47	2.16	2.05 – 2.24	0.5%	0.4%
60 – 69	13,004	0.44	0.42 – 0.46	2.23	2.14 – 2.32	0.5%	0.5%
70 – 79	6,807	0.44	0.39 – 0.47	2.39	1.92 – 2.54	0.5%	0.7%
≥80	1,894	0.50	0.44 – 0.54	3.99	2.54 – 9.62	0.2%	1.4%
eGFR, mL/min/1.73 m^2							
60 – 89	27,420	0.46	0.45 – 0.48	2.31	2.20 – 2.39	0.4%	0.6%
≥90	14,462	0.42	0.41 – 0.45	2.15	2.06 – 2.24	0.6%	0.4%

Standard reference intervals: kappa 3.3 – 19.4 mg/L, lambda 5.7 – 26.3 mg/L and FLC ratio 0.26 – 1.65. Abbreviations: eGFR, estimated glomerular filtration rate; FLC, free light chain.

© 2025 Einarsson Long T et al. *JAMA Oncology*.

eTable 2. Rate of Abnormal Results in Age, Sex, and eGFR Subgroups When a Revised Reference Interval for Kappa FLC, Lambda FLC, and the FLC Ratio is Used in Persons Below and Above Age 70 Years

Kappa FLC, mg/L			
Group	N	% below reference	% above reference
**Age <70 years**	33181		
Females	19300	0.6%	0.4%
Males	13881	0.4%	0.7%
Age, years			
40 – 49	7389	0.7%	0.3%
50 – 59	12788	0.5%	0.4%
60 – 69	13004	0.4%	0.7%
eGFR,mL/min/1.73 m^2			
60-89	19261	0.3%	0.6%
≥90	13920	0.8%	0.4%
**Age ≥70 years**	8701		
Females	4486	0.7%	0.4%
Males	4215	0.3%	0.6%
Age, years			
70 – 79	6807	0.5%	0.4%
≥80	1894	0.4%	0.7%
eGFR,mL/min/1.73 m^2			
60 – 89	8159	0.4%	0.5%
≥90	542	1.7%	1.1%
**Lambda FLC, mg/L**			
Group		% below reference	% above reference
**Age <70 years**	33181		
Females	19300	0.4%	0.4%
Males	13881	0.6%	0.6%
Age, years			
40 – 49	7389	0.6%	0.4%
50 – 59	12788	0.5%	0.4%
60 – 69	13004	0.4%	0.6%
eGFR (mL/min/1.73 m^2)			
60 – 89	19261	0.4%	0.5%
≥90	13920	0.6%	0.5%
**Age ≥70 years**	8701		
Females	4486	0.6%	0.4%
Males	4215	0.4%	0.7%
Age, years			
70 – 79	6807	0.4%	0.5%
≥80	1894	0.6%	0.5%
eGFR,mL/min/1.73 m^2			
60 – 89	8159	0.5%	0.5%
≥90	542	0.6%	0.6%

© 2025 Einarsson Long T et al. *JAMA Oncology*.

FLC ratio<br>Group		% below reference	% above reference
**Age <70 years**	33181		
Females	19300	0.5%	0.4%
Males	13881	0.5%	0.7%
Age, years			
40 – 49	7389	0.5%	0.4%
50 – 59	12788	0.5%	0.5%
60 – 69	13004	0.5%	0.6%
eGFR,mL/min/1.73 m^2)			
60 – 89	19261	0.4%	0.5%
≥90	13920	0.6%	0.5%
**Age ≥70 years**	8701		
Females	4486	0.5%	0.5%
Males	4215	0.6%	0.5%
Age, years			
70 – 79	6807	0.6%	0.4%
≥80	1894	0.3%	0.8%
eGFR,mL/min/1.73 m^2			
60 – 89	8159	0.5%	0.5%
≥90	542	1.1%	0.4%

Abbreviations: eGFR, estimated glomerular filtration rate; FLC, free light chain.

© 2025 Einarsson Long T et al. *JAMA Oncology*.

eTable 3. Revised Central 95% Reference Intervals for Serum Kappa FLC, Lambda FLC and FLC Ratio in Individuals With Preserved Kidney Function (eGFR ≥60 mL/min/1.73 m^2), Stratified by Age

Kappa FLC, mg/L					
Group	N	2.5th percentile		97.5th percentile	
		Kappa, mg/L	95% CI	Kappa, mg/L	95% CI
Age < 70 years	33,181	7.7	7.6 – 7.7	27.4	27.0 – 27.7
Age ≥70 years	8,701	8.6	8.4 – 8.7	35.6	34.8 – 36.9
**Lambda FLC, mg/L**					
Group	N	2.5th percentile		97.5th percentile	
		Lambda, mg/L	95% CI	Lambda, mg/L	95% CI
Age <70 years	33,181	7.5	7.5 – 7.6	27.1	26.8 – 27.4
Age ≥70 years	8,701	8.1	8.0 – 8.3	31.5	30.6 – 32.4
**FLC ratio**					
Group	N	2.5th percentile		97.5th percentile	
		FLC ratio	95% CI	FLC ratio	95% CI
Age <70 years	33,181	0.57	0.57 – 0.58	1.69	1.68 – 1.71
Age ≥70 years	8,701	0.61	0.60 – 0.63	1.84	1.81 – 1.88

Standard reference intervals: kappa 3.3 – 19.4 mg/L, lambda 5.7 – 26.3 mg/L and FLC ratio 0.26 – 1.65. Abbreviations: FLC, free light chain.

© 2025 Einarsson Long T et al. *JAMA Oncology*.

eTable 4. Characteristics of the Persons Excluded From the Determination of FLC Reference Intervals

	Overall	<70 years	≥70 years
N	33540	23688	9828
Age, years	63 (54-72)	58 (51-64)	77 (74-82)
Sex, males	16093 (48.0)	11219 (47.4)	4863 (49.5)
eGFR, mL/min/1.73 m^2	78 (57-92)	86 (72-97)	57 (47-72)
Kappa FLC, mg/L	15.4 (12.2-20.5)	14.1 (11.6-17.6)	20.8 (15.8-28.8)
Lambda FLC, mg/L	14.6 (11.6-18.8)	13.6 (11.1-16.9)	17.9 (13.9-23.8)
FLC ratio	1.09 (0.90-1.31)	1.05 (0.88-1.26)	1.18 (0.96-1.44)
Comorbidities			
Hypertension	12324 (38.3)	6051 (27.0)	6261 (64.7)
Diabetes	2288 (7.1)	1031 (4.6)	1254 (13.0)
Ischemic heart disease	4326 (13.5)	1492 (6.6)	2826 (29.2)
Heart failure	1657 (5.2)	356 (1.6)	1300 (13.4)
Cardiac arrythmia	3911 (12.2)	1414 (6.3)	2494 (25.8)
Peripheral artery disease	1087 (3.4)	399 (1.8)	687 (7.1)
Obesity	2474 (7.7)	1736 (7.7)	736 (7.6)
Endocrine disorder (non-diabetic)	3776 (11.7)	2204 (9.8)	1568 (16.2)
Chronic lung disease	8728 (27.2)	5229 (23.3)	3498 (36.2)
Liver disease	543 (1.7)	311 (1.4)	232 (2.4)
Malignancy	3698 (11.5)	1608 (7.2)	2088 (21.6)
Neurological disease	1056 (3.3)	534 (2.4)	520 (5.4)

Data are presented as number (%) for categorical variables and median (interquartile range) for continuous variables.

Abbreviations: eGFR, estimated glomerular filtration rate; FLC, free light chain.

© 2025 Einarsson Long T et al. *JAMA Oncology*.

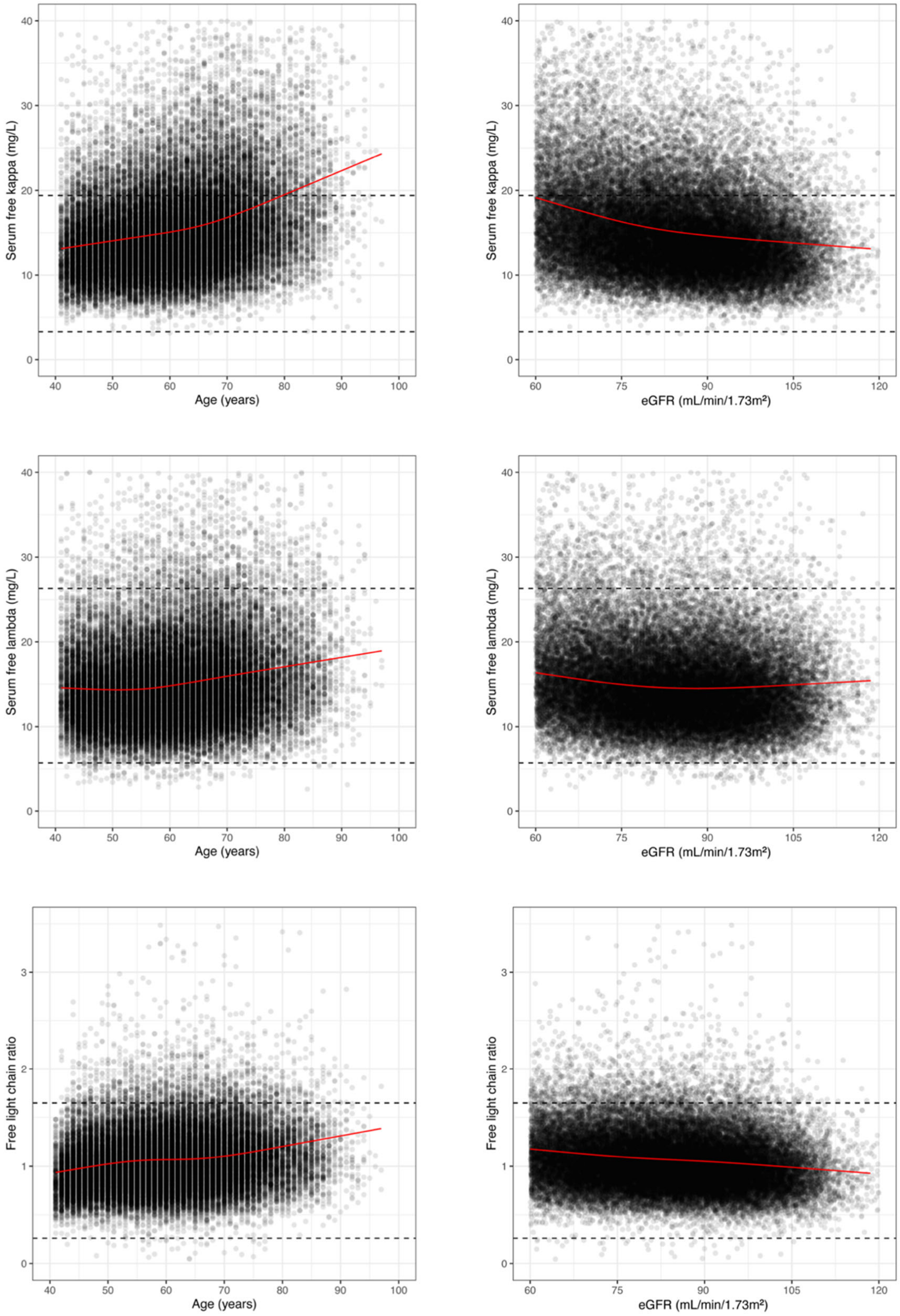

eFigure. Scatterplots Demonstrating the Association of Serum Kappa FLC, Lambda FLC and the FLC Ratio With Age and eGFR

The red line demonstrates restricted cubic splines of serum kappa FLC, lambda FLC and FLC ratio with age and eGFR. Dashed lines show standard reference intervals. The y axis is truncated at 45 mg/L for better visualization. eGFR, estimated glomerular filtration rate; FLC, free light chain.

© 2025 Einarsson Long T et al. *JAMA Oncology*.
