# Supplementary material for: New Definition of Light Chain Monoclonal Gammopathy of Undetermined Significance
Source: JAMA Oncol. 2025 May 29;11(7):753–61. doi: 10.1001/jamaoncol.2025.1285 (PMC12123532; doi:10.1001/jamaoncol.2025.1285)
Supplement: Supplement 2. — Data Sharing Statement [file jamaoncol-e251285-s002.pdf]

Data Sharing Statement

Einarsson Long. New Definition of Light Chain Monoclonal Gammopathy of Undetermined Significance. *JAMA Oncol*. Published May 29, 2025. doi:10.1001/jamaoncol.2025.1285

Data

Data available: Yes
Data types: Other (please specify)
Additional Information: Statistical code and data set will be available from the corresponding author on reasonable request. Guidelines on data availability will be followed as long as they are in line with our IRB and Data Protection Authority permits.
How to access data: For requesting access to the study data please contact the corresponding author at thorirein@gmail.com or the princpal investigator at sigyngvi@hi.is.
When available: With publication

Supporting Documents

Document types: Statistical/analytic code, Informed consent form
How to access documents: For requesting access to the statistical code please contact the corresponding author at thorirein@gmail.com. For access to the informed consent form please contact the principal investigator at sigyngvi@hi.is
When available: With publication

Additional Information

Who can access the data: Statistical code and data set will be available from the corresponding author on reasonable request. Guidelines on data availability will be followed as long as they are in line with our IRB and Data Protection Authority permits.
Types of analyses: The data will be made available for any purpose of scientific or patient value as they are in line with our IRB and Data Protection Authority permits.
Mechanisms of data availability: THe data will be made available with investigator support
